# Vasopressin Differentially Affects Handgrip Force of Expectant Fathers in Reaction to Own and Unknown Infant Faces

**DOI:** 10.3389/fnbeh.2019.00105

**Published:** 2019-05-21

**Authors:** Kim Alyousefi-van Dijk, Anna E. van ‘t Veer, Willemijn M. Meijer, Anna M. Lotz, Jolien Rijlaarsdam, Jurriaan Witteman, Marian J. Bakermans-Kranenburg

**Affiliations:** ^1^Clinical Child and Family Studies, Faculty of Behavioral and Movement Sciences, Vrije Universiteit, Amsterdam, Netherlands; ^2^Leiden Institute for Brain and Cognition, Leiden University Medical Center, Leiden, Netherlands; ^3^Methodology and Statistics Unit, Institute of Psychology, Faculty of Social and Behavioural Sciences, Leiden University, Leiden, Netherlands; ^4^Department of Child and Adolescent Psychiatry/Psychology, Erasmus MC-University Medical Center Rotterdam, Rotterdam, Netherlands; ^5^Leiden University Centre for Linguistics, Leiden University, Leiden, Netherlands

**Keywords:** paternal care, vasopressin, handgrip paradigm, infant crying, facial resemblance

## Abstract

The underlying mechanisms of paternal responses to infant signals are poorly understood. Vasopressin has previously been proposed to affect these responses. Using a double-blind, placebo-controlled, within-subject design (*N* = 25 expectant fathers), we examined the effect of vasopressin administration on the use of excessive handgrip force during exposure to infant crying versus matched control sounds, while participants saw morphed images representing their own infant versus an unknown infant. We found that, compared to placebo, AVP administration elicited more excessive force while viewing an unknown infant image compared to viewing the image representing one’s own infant, while the reverse was true under placebo. The results are discussed in light of vasopressin’s role in parenting and parental protection among human fathers.

## Introduction

Infant crying can evoke parental proximity and care (Bowlby, 1969/1982; Groh and Roisman, 2009), yet it is also an aversive stimulus (Murray, 1985; Fujiwara et al., 2011; Leerkes et al., 2011) with the potential to trigger child abuse, neglect and infanticide (e.g., Soltis, 2004; Out et al., 2012). Infant crying is a highly salient cue that results in physiological arousal in both females and males (Frodi and Lamb, 1978; Groh and Roisman, 2009) and is particularly potent in activating the parental caregiving system (George and Solomon, 2008). Individual variation in the physiological response to infant crying has previously been proposed to play a role in the behavioral response to these infant signals (e.g., Murray, 1985; Reijman et al., 2016). Although several researchers have studied the physiological response to infant crying in females, both with and without children of their own (e.g., Bugental et al., 1999; Bakermans-Kranenburg et al., 2012; Riem et al., 2012, 2016; Compier-de Block et al., 2015), the mechanisms involved in the reactions of males to infant crying remain largely unknown. At the same time, males represent about 50% of all parents, and they actively participate in parenting at increases rates, making insight into the mechanisms involved in reactions of males to infant crying urgent. In particular, the need to examine the role of hormones, especially vasopressin (AVP), in paternal behavior has recently been stressed (Rilling and Mascaro, 2017). The current study investigates whether AVP affects the use of handgrip force in reaction to infant crying while exposed to images representing one’s own as well as an unknown infant in expectant fathers.

The handgrip dynamometer paradigm has previously been used as a behavioral measure to study responses to infant crying. This paradigm measures the degree to which someone does not have control over handgrip force, and excessive handgrip force is used as an indicator of physiological hyperactivity and/or a lack of control over autonomic responses. For example, parents at risk for child abuse—compared to low risk parents—are found to not only rate videos of crying infants more negatively and report higher levels of hostile feelings, they also use more excessive handgrip force after hostile priming than their low risk counterparts (Crouch et al., 2008). Likewise, in response to crying as well as laughter, maltreating mothers use more excessive handgrip force than non-maltreating mothers (Compier-de Block et al., 2015). Several factors have been shown to relate to the use of excessive handgrip force in pseudo-parenting contexts. For example, compared to other women, mothers with low perceived parental control have been found to show heightened autonomic arousal and more use of excessive force in operating the dynamometer while providing negative feedback to children who show ambiguous behavior (Bugental et al., 1999). Additionally, females with insecure attachment representations have been found to experience more irritation during infant crying and use more excessive handgrip force than females with secure attachment representations (Riem et al., 2012). Due to the association with maladaptive parenting, studying the processes underlying excessive handgrip force may help find physiological markers of parenting style in general.

Throughout evolution, human fathers have been faced with paternity uncertainty (Buss, 1999; Larsen and Buss, 2009) and have used cues of genetic relatedness, such as facial resemblance, in allocating offspring investment (e.g., Apicella and Marlowe, 2004; Alvergne et al., 2009; DeBruine et al., 2009; Yu et al., 2017, 2019). This strategy maximizes reproductive benefits as limited resources are preferentially assigned to offspring passing down fathers’ genes to next generations (Kaplan and Gangestad, 2005; Griskevicius et al., 2011). When paternal care is provided, offspring has a higher chance of survival and a better quality of life (e.g., Hurtado and Hill, 1992; Flouri and Buchanan, 2003, 2004; Sear and Mace, 2008), even in modern societies (Vågerö et al., 1998). Likewise, men, rather than women, have been found to base a hypothetical adoption choice primarily on cues of kinship (Volk and Quinsey, 2002) and the self-reported quality of fathers’ relationship with their children is predicted by the level of resemblance (Apicella and Marlowe, 2004). Naturally, human behavior is not exclusively shaped by evolutionary processes but also by social and cultural contexts (Shan et al., 2012; Bertamini and Lyons, 2015; Yu et al., 2017), as is evident by the findings of Abraham et al. (2014) indicating there are no differences in the neural processing of viewing one’s own infant by either biological or adoptive fathers. Nonetheless, with the exception of adoptive fathers (Van IJzendoorn et al., 2009), parents tend to invest less in biologically unrelated versus related children (e.g., Alvergne et al., 2009). Moreover, Daly and Wilson (1984) found that males who had committed infanticide commonly report an absence of resemblance with the child in question [but see Temrin et al. (2000) and Daly and Wilson (2008) for a debate on the effects of these evolutionary processes in modern society]. Although not previously investigated, handgrip force (or a lack of control thereof) during exposure to resembling and non-resembling infant images could potentially highlight the evolutionary beneficial distinction commonly found in males since resources (e.g., time and energy spend on suppressing the aversiveness of infant crying in order to provide adequate parental care) are preferentially allocated to the own infant at the expense of unrelated infants. Combining the discussed findings on abuse with the fact that infant crying is a common trigger for child abuse and infanticide (see Zeifman and St James-Roberts, 2017 for a review), it is likely that there are differential physiological mechanisms at play when an aversive and highly salient stimulus such a as crying is coupled with the image of an infant that is likely versus unlikely related to the observing male, particularly in periods of changing hormone levels and reactivity.

Recent studies suggest that handgrip force in reaction to infant signals is influenced by hormones: administration of oxytocin (OT) reduces the use of excessive force in insecurely attached females (Riem et al., 2016), and in females without a history of harsh discipline (Bakermans-Kranenburg et al., 2012). Furthermore, Riem et al. (2017) showed that in males, an experimental increase in endogenous OT release through mechanically delivered massage is related to reduced handgrip force during exposure to infant sounds. These results suggest that hormones play an important role in the physiological response to infant crying in both females and males. Next to OT, AVP is also considered to be functionally significant in parental behavior (Wang et al., 2000; Ahern and Young, 2009; Ahern et al., 2010; Gouin et al., 2010; Snowdon et al., 2010; Apter-Levi et al., 2014). Specifically, AVP is suggested to be more strongly related to paternal parenting than to maternal parenting (Wang et al., 2000; Wynne-Edwards, 2001; Storm and Tecott, 2005; Carter et al., 2007; Taylor et al., 2010). Additionally, when it comes to pair bonding, AVP has been proposed to fulfill a similar role in males as OT in females (Taylor et al., 2000, 2010). However, its exact role in paternal care remains largely unclear, making AVP an obvious candidate for closer examination in the context of how males respond to infant signals.

Sexually dimorphic roles of AVP have been found in both humans (e.g., Thompson et al., 2006) and animals (e.g., see Terranova et al., 2017). For example, in response to watching an interaction with one’s own child, activations in social-cognitive circuits were correlated with fathers’ but not mothers’ salivary AVP levels (Atzil et al., 2012). Additionally, the male mandarin vole has been found to show differential behavioral, hormonal, and neural responses to own versus unknown pups, with AVP neurons specifically responding to the voles’ own pups (Yuan et al., 2018). Considering its crucial role in protective aggression as a part of paternal care (Van Anders et al., 2011; Bakermans-Kranenburg and Van IJzendoorn, 2017), it is likely that paternal AVP plays a role in the physiological response and emotional regulation when exposed to infant crying (Meyer-Lindenberg et al., 2011). Specifically, AVP might be implicated in making the evolutionary beneficial distinction between one’s own versus unrelated offspring. Indeed, in rodents, AVP is associated not only with male bonding, defensive and territorial behavior (Bielsky et al., 2005), but also with social recognition (Caldwell et al., 2008). In humans, AVP has also been found to play an important role in social recognition and face perception (Guastella et al., 2010). For instance, AVP-dependent paternal brain activations and hormonal responses have been suggested to underlie fathers’ ability to interpret others’ intentions in order to accurately defend offspring (Thompson et al., 2006; Atzil et al., 2012). By fostering selective protection, AVP may facilitate successful fathering in humans and other mammals (Carter, 1998). The effect of AVP on responses to infant cries may therefore depend on the presence of kinship cues. Additionally, AVP administration increases salience processing in the brain (Brunnlieb et al., 2013; Feng et al., 2015) and can therefore be expected to play a role in highlighting the salience of infant cry signals, particularly in periods with marked AVP sensitivity.

Expectant fathers can undergo physical changes that prepare them for fatherhood, which are, at least in part, due to changes in various hormone levels (see Wynne-Edwards, 2001). Coinciding with an increase in caregiving behaviors and caregiving attitudes in expectant fathers (Cohen-Bendahan et al., 2015), robust changes in prolactin, cortisol and sex steroids have been found during the prenatal period (e.g., Storey et al., 2000; Berg and Wynne-Edwards, 2001; Edelstein et al., 2015). Also, basal levels of testosterone and cortisol, as well as prolactin have been shown to relate to responsiveness to infants and to prenatal quality of caregiving in expectant fathers (Storey et al., 2000; Bos et al., 2018). One study found that although basal levels of OT and AVP are no different in fathers-to-be compared to non-expecting men, administration of AVP to fathers-to-be results in an increase of parenting behaviors whereas administration to non-expecting males does not (Cohen-Bendahan et al., 2015). These findings suggest an increase of sensitivity to AVP in fathers during the prenatal period.

To our knowledge, the current exploratory study is the first to examine whether, in expectant fathers, administration of AVP affects the use of excessive handgrip force in response to infant crying paired with images representing one’s own or an unknown infant. To this end, a double-blind, placebo-controlled, within-subject design was used. On the basis of the literature described here, it can be expected that fathers-to-be would use more excessive force during cry sounds (i.e., a salient and aversive stimuli) compared to neutral sounds and more excessive force while viewing an unknown versus their own infant under crying conditions. Considering the limited literature on the effects of AVP in expectant fathers, we examined if AVP administration would differentially affect these responses. Considering findings indicating that experienced caregiving in parents’ own childhood alter performance on a parenting-related handgrip task (Bakermans-Kranenburg et al., 2012) as well as neural responses to cry sounds after AVP administration (Thijssen et al., 2018) we will take these experiences into account.

## Materials and Methods

### Participants

Twenty-five first-time expectant fathers were recruited through midwives and ads on Leiden University affiliated webpages. Sample size was determined by the ethics approval request for a first study with AVP administration in our lab. Participants cohabitated with their pregnant partners, spoke Dutch, and were screened and excluded for self-reported neurological, neuroendocrine and psychiatric disorders, and alcohol and substance abuse. The mean age of the participants was 31.92 years (*SD* = 4.30). The mean gestational age of the unborn infants was 27.02 weeks (*SD* = 4.91). See Table 1 for information on demographics. This experiment was part of a larger study (see also Thijssen et al., 2018; Van ’t Veer et al., 2019). Permission for this study was obtained from the Ethics Committees of the Institute for Education and Child Studies at Leiden University and the Leiden University Medical Centre, as well as the Dutch Central Committee on Research Involving Human Subjects. All participants gave informed consent.

**TABLE 1:** Demographic information of the sample (*N* = 25).

		***M*(*SD*)/*N*(%)**	**Min**	**Max**
Age (years)		31.92 (4.30)	24.65	43.04
Gestational age (weeks)		27.02 (4.91)	20.43	36.14
Education	Secondary	5 (20%)		
	Higher	20 (80%)		
Income	< €3200	6 (24%)		
	€3200 – €4000	10 (40%)		
	> €4000	9 (36%)		
Handedness	Right handed	23 (92%)		
Condition session 1	AVP	13 (52%)		
Time between nasal spray and handgrip paradigm (min)	Placebo	126 (9)	1:53	2:30
	AVP	125 (8)	1:52	2:28

### Procedure

Participants visited the lab twice with an intervening period of 1 week. They self-administered a single dose of 20 IU AVP or placebo nasal spray at the start of each session (counterbalanced) using a syringe with a MAD Nasal^TM^ Device. Dosage was chosen based on previous studies of AVP effects on behavior and brain activation (e.g., Pitman et al., 1993; Thompson et al., 2006; Rilling et al., 2012; Uzefovsky et al., 2012; Tabak et al., 2015). This dose has been found to result in elevations in plasma equivalent to those produced by an intravenous dose of 0.025 IU (Pietrowsky et al., 1996). After administration participants first completed tasks in an MRI scanner (i.e., a resting state scan, a working memory task, a task involving labeled cry sounds, see Thijssen et al., 2018, and a task with video vignettes, see Van ’t Veer et al., 2019), followed by three behavioral tasks, starting with the handgrip paradigm described here. On average the handgrip paradigm took place 125.5 min (*SD* = 8.5) after nasal spray administration. Of the 24 participants who answered the question about which substance they thought they were given during the second session (placebo, AVP, or unsure), only 10 were correct. In the week between the two sessions, participants filled out online questionnaires at home including the Conflict Tactics Scale (CTS, Straus et al., 1998) and a subscale of the Children’s Report of Parental Behavior Inventory (CRBI, as used in Huffmeijer et al., 2011), which serve as our measures of early caregiving experiences of the fathers-to-be.

### Measures

#### Handgrip Dynamometer

Participants were exposed to infant crying and images representing either their own or an unknown infant while they were asked to squeeze a handgrip dynamometer (similar to Bakermans-Kranenburg et al., 2012).

In order to create suitable images for the handgrip paradigm, participants either provided a full-color digital photograph of themselves prior to the first session, or a picture was taken at the beginning of the first visit. The participant’s picture met the following criteria: it showed their face, en face, with a neutral expression, a light and neutral background, without piercings, make-up, or glasses. Photographs were edited using Adobe Photoshop CS in order to remove unwanted facial features (e.g., facial hair). Subsequently, morphed images representing participant’s own infant were created by combining 75% of an average infant image (created by the authors of Hahn et al., 2015, from 10 female and 10 male infant faces) and 25% of participant’s own picture, using Fantamorph 5 Deluxe^1^. Similarly, the morphed image of an unknown infant was created by combining 75% of the average infant image and 25% of a male unknown to the participants, after which all images were resized to 640 × 480 pixels. Finally, images were masked with a black face contour. Participants were familiarized with their morphed own infant image before onset of the task with the explanation that a future infant of theirs might look similar to this image. The same own and unknown infant images were also used during one of the fMRI tasks (see Van ’t Veer et al., 2019) prior to the handgrip paradigm. During this fMRI task, and contrary to the handgrip paradigm, the images were presented alongside text inviting the participants to imagine seeing their own or an unknown infant, respectively.

A total of three cry sounds were used from two infants, one male (two sounds) and one female (one sound) recorded with a TasCam DR-05 solid state recorder with a 44.1 Khz sampling rate and 16 bit. All sounds were recorded within the first two prenatal days. Individual sounds were scaled, the intensity was normalized to the same mean intensity and sounds were edited using PRAAT software (Boersma and Weenink, 2017). For each cry sound a neutral auditory control stimulus was created by calculating the average spectral density over the entire duration of the original sound. A continuous sound of equal duration was re-synthesized from the average spectral density and amplitude modulated by the amplitude envelope, extracted from the original sound. After this procedure, all auditory stimuli and control stimuli were intensity matched. Using this procedure, the neutral auditory control stimuli were identical to the original auditory stimuli in terms of duration, intensity, spectral content, and amplitude envelope, but lacking the emotional meaning associated with a cry sound.

During the task participants were seated comfortably in front of a computer screen wearing headphones while holding a dynamometer in their dominant hand. During an initial training period, participants were asked to squeeze the handgrip dynamometer at full and half strength while they received feedback from a monitor indicating the strength they used graphically. Once participants could reliably alternate between full and half strength (half strength being 50% of the strength used at full strength), the actual task began in which participants received no further feedback on their performance.

The task was administered using E-Prime software (version 2.0; Psychology Software Tools, Inc., Sharpsburg, PA, United States) and hand squeeze intensities (in kg) were transferred directly from the dynamometer to AcqKnowledge software (version 4.3.1; Biopac Systems, 2004). First, a baseline measure of three maximum strength trials each followed by half strength trials was administered. Then, four randomly presented conditions of three max-half trials were presented; (1) viewing a morphed image of own infant while hearing control (scrambled) sounds (Own Neutral); (2) viewing a morphed image of own infant while hearing cry sounds (Own Cry); (3) viewing a morphed image of an unknown infant while hearing control (scrambled) sounds (Other Neutral); (4) viewing a morphed image of an unknown infant while hearing cry sounds (Other Cry). Handgrip force measures were reliable (α = 0.75 – 0.89) in all four conditions consisting of three trials each, across both placebo and AVP sessions. Therefore, the three trials per condition were averaged as an indicator of handgrip force in each condition. Sounds and images were presented throughout each trial lasting 12 s. Eight seconds after the beginning of each trial, participants were prompted to squeeze maximally (instructions displayed for 1 s). After an interval of 2 s, participants were prompted to squeeze at half strength (instructions were displayed for 1 s). A fixation cross was shown for 3 s between each trial.

Similar to previous studies (Bakermans-Kranenburg et al., 2012; Riem et al., 2012; Compier-de Block et al., 2015), grip strength modulation was calculated by dividing half-strength squeeze intensity by the preceding full-strength squeeze intensity, meaning that scores of over 0.50 indicated excessive force on the half-strength squeeze attempt. We examined the effects of AVP on this ratio of handgrip strength at half force and handgrip strength at maximum force, which can be considered an implicit measurement of reactive force in response to infant signals. Matlab (version 8.0.0.783, Mathworks, Natick, MA, United States) was used to identify peak intensities for each squeeze.

#### Mood

In order to assess possible mood induction by the preceding fMRI tasks and a potentially differential effect of hormone administration, the current emotional status of participants was measured using the Positive and Negative Affect Schedule (PANAS; Watson et al., 1988) in between MRI and behavioral measurements.

#### Associations With Early Caregiving Experiences

Early caregiving experiences were considered to be possible related to our outcome measures. Participants completed the Conflict Tactics Scale – Parent Child (CTS, Straus et al., 1998), which assesses experienced abuse and neglect during the participants’ childhood, and the Love Withdrawal subscale of the Children’s Report of Parental Behavior Inventory (CRPBI, Schludermann and Schludermann, 1983; Beyers and Goossens, 2003), see Supplementary Material for details of these measures.

## Results

Positive mood scores were roughly the same in the AVP (*M* = 32.04, *SD* = 6.56) and placebo (*M* = 31.96, *SD* = 7.52) conditions. Similarly, negative mood scores were roughly the same in the AVP (*M* = 13.96, *SD* = 3.63) and placebo (*M* = 13.32, *SD* = 2.98) conditions. Measures of early caregiving experiences were not found to correlate strongly with mean handgrip force ratio’s (see Supplementary Material). Under baseline conditions (i.e., no sounds or images presented), as well as in all other conditions, the mean handgrip force ratio was just above the instructed 0.5, see Table 2. Mean handgrip force ratio during the AVP baseline was slightly lower than handgrip force ratio during placebo baseline, see Table 2.

**TABLE 2:** Mean handgrip force ratio’s for all conditions, calculated by the ratio between half strength and maximum strength.

	***M*(*SD*)**
Placebo, baseline	0.63 (0.11)
AVP, baseline	0.58 (0.09)
Placebo, own infant, control sound	0.63 (0.12)
AVP, own infant, control sound	0.63 (0.14)
Placebo, own infant, cry sound	0.66 (0.13)
AVP, own infant, cry sound	0.64 (0.13)
Placebo, unknown infant, control sound	0.62 (0.15)
AVP, unknown infant, control sound	0.66 (0.13)
Placebo, unknown infant, cry sound	0.62 (0.13)
AVP, unknown infant, cry sound	0.66 (0.14)

In order to examine the patterns in the data for the effect of AVP on the use of excessive handgrip force while listening to infant cry sounds as well as viewing one’s own and an unknown infant images, a repeated measures analysis of variance with condition (AVP versus placebo), sound (cry versus control sounds), and familiarity (unknown versus own infant image) as factors and mean handgrip force ratio as the dependent variable was conducted. A Shapiro–Wilk test indicated that all data was normally distributed (*p* > 0.1 for all conditions).

Results did not show main effects of condition (AVP versus placebo, *F*[1,24] = 0.68, *p* = 0.42, *$\eta_{p}^{2}$* = 0.03), sound (cry versus control sounds, *F*[1,24] = 1.46, *p* = 0.24, *$\eta_{p}^{2}$* = 0.05), or familiarity (unknown versus own infant image, *F*[1,24] = 0.00, *p* = 0.98, *$\eta_{p}^{2}$* = 0.00).

**FIGURE 1:**
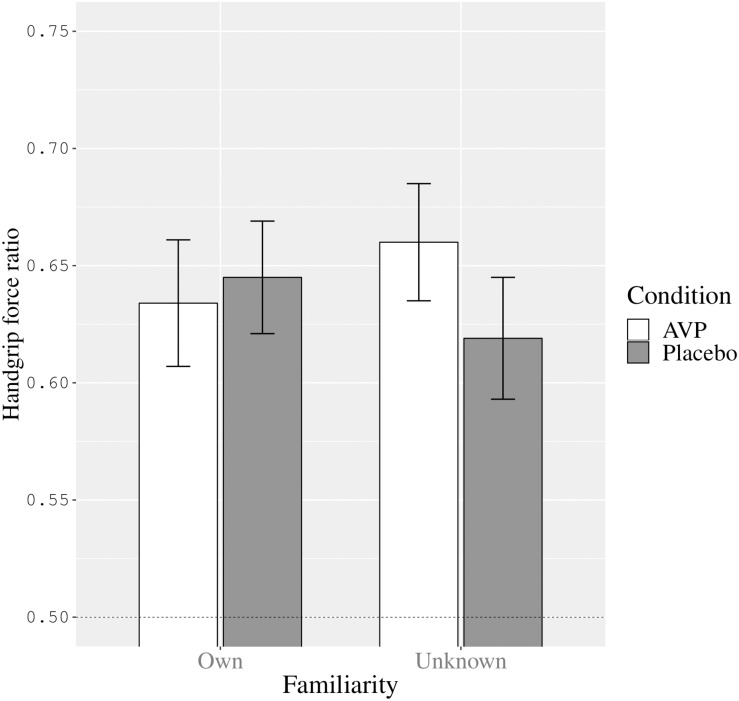
The interaction between condition (AVP versus placebo) and familiarity (own versus unknown infant image) on participants’ handgrip force ratio (Estimated marginal means and Standard errors). The dashed line represents control over handgrip force in line with the instructions to first squeeze as hard as possible and then squeeze at half that strength (corresponding to a handgrip force ratio of 0.50).

However, the two-way interaction between condition and familiarity was significant (*F*[1,24] = 6.27, *p* = 0.02, $\eta_{p}^{2}$ = 0.21). We present the estimated marginal means and standard errors for this interaction in Figure 1. Compared to placebo, AVP had a differential effect on handgrip force while watching one’s own versus an unknown infant. As can be seen in Figure 1, the interaction between condition and familiarity was such that, in contrast with placebo, AVP administration elicited more excessive force while viewing an unknown infant image compared to viewing one’s own infant’s image, while the reverse was true for placebo administration. In other words, under AVP there was less excessive handgrip force while viewing an image representing one’s own infant compared to while viewing an unknown infant.

Upon reviewer suggestion we also ran *post hoc t*-tests on the familiarity means within each condition separately. This revealed that, with medium effect size, while viewing the unknown infant image participants tended to used more excessive handgrip force in the vasopressin condition (*M* = 0.66, *SE* = 0.03) compared to placebo condition (*M* = 0.62, *SE* = 0.03), *F*(1,24) = 3.51, *p* = 0.07, $\eta_{p}^{2}$ = 0.13, 95% CI [−0.004,0.086]. In contrast, while viewing one’s own infant image, there was not such a difference in handgrip force in the vasopressin condition (*M* = 0.63, *SE* = 0.03) and the placebo condition (*M* = 0.65, *SE* = 0.02), *F*(1,24) = 0.32, *p* = 0.58, $\eta_{p}^{2}$ = 0.01, 95% CI [−0.05,0.03].

Results did not show a significant two-way interaction effect between condition and sound (*F*[1,24] = 0.17, *p* = 0.68,$\eta_{p}^{2}$ = 0.01), nor between infant and sound (*F*[1,24] = 0.62, *p* = 0.44, *$\eta_{p}^{2}$* = 0.03). The three-way interaction between condition, sound, and familiarity was not significant either (*F*[1,24] = 0.95, *p* = 0.34, *$\eta_{p}^{2}$* = 0.04).

## Discussion

In a randomized controlled within-subject experiment we tested the effect of AVP administration on the use of excessive handgrip force during exposure to infant crying and images of one’s own versus an unknown infant in fathers expecting their first child. Contrary to our expectation, expectant fathers did not use significantly more handgrip force during infant cry sounds versus control sounds. Results indicated that AVP administration affected the use of handgrip strength differently depending on whether a morphed image representing participant’s own infant or an unknown infant was shown. More specifically, compared to placebo, fathers used more handgrip force under AVP when looking at an image representing an unknown infant than when looking at an image representing their own infant, while under placebo it was the other way around. That is, under AVP the mean ratio of handgrip force (a proxy for control over autonomic responses) increased while viewing and unknown infant but decreased while viewing one’s own infant. These findings were independent of the accompanying sound (i.e., cry versus control sounds).

The follow-up analyses illustrating and exploring the interaction effect of hormone administration and image showed that compared to placebo, the administration of AVP in expectant fathers increased handgrip force in response to an unknown (but not own) infant. This finding is in line with indications from previous literature. Long known from clinical and behavioral studies, men are more likely to provide care for biologically related children and less likely to abuse them compared to unrelated children (e.g., Daly and Wilson, 1988; Burch and Gallup, 2000), with the exception of adoptive fathers (Van IJzendoorn et al., 2009). Assessing paternal resemblance, starting soon after birth, has been shown to be an important mechanism in estimating the chances of paternity (Daly and Wilson, 1982, 1998; Lacy and Sherman, 1983). Interestingly, even the unconscious determination of self-resemblance in a child’s face may affect attitudes and intended care toward the child. For instance, studies have found that morphed child images bearing resemblance to participants were rated as more attractive, were more likely to be adopted in a hypothetical situation, and received more hypothetical time and money by male (but not female) participants (Platek et al., 2002, 2004; but see DeBruine, 2004 for methodological considerations on the gender difference for these effects). This idea is supported by findings that males show differential brain responses to resembling versus non-resembling child images, supposedly underlying either heightened attention allocated to assess resemblance, or increased reward related activation when the child is likely to be genetically related (e.g., Platek et al., 2005, 2008). AVP administration may magnify such differentiating processes. Whether the differential effect of AVP on reactions to own versus unknown infants was based on conscious or unconscious recognition of the images cannot be determined since participants were not asked to identify or rate the images.

Additionally, our findings are in line with previous suggestions proposing that changes in sensitivity to AVP during the prenatal period, and changes in AVP expression postnatally, promote paternal behavior (e.g., see Gettler, 2014 resp.; Cohen-Bendahan et al., 2015). In accordance with the Steroid/Peptide Theory of Social Bonds (Van Anders et al., 2011) this study supports the notion that AVP may be involved in a critically important but understudied part of paternal care, namely protective aggression. Our finding concerning the use of more excessive force while viewing an unknown versus one’s own infant image may speculatively be related to an increase in protective parenting behaviors induced by the administration of AVP. Under such circumstances, the evolutionary beneficial recognition of related offspring could result in preferential allocation of resources to their own infant rather than a unrelated infant. The presence of aversive and now increasingly salient infant crying could have prompted our expectant fathers to relatively high levels of intolerance for crying unrelated infants. Future studies can further parse out whether AVP indeed underlies mechanisms of protective aggressive responses to non-kin in expectant fathers.

We did not find a significant effect of cry sounds (versus control sounds) on fathers’ handgrip force. Even though exposure to infant crying (versus control sounds) increased activation in the bilateral auditory cortex and posterior medial cortex in the same group of participants (Thijssen et al., 2018), these effects do not seem to relate to behavioral change as measured in the present study by the handgrip paradigm outside the scanner. In accordance with the results presented here, Thijssen et al. (2018) found no effect of AVP on these neural responses to infant crying. However, AVP did selectively affect the neural processing of infant cries coupled with an emotional context label (e.g., ‘this infant is sick’) compared to cry sounds coupled with a neutral label (e.g., ‘this is an infant’). Effects of AVP can be highly context-dependent (Van Anders et al., 2011; Gettler, 2014), and real-life cry sounds are usually coupled with contextual information that may push caretakers toward protective or caretaking behavior. The absence of contextual information accompanying the cry sounds presented in the handgrip paradigm may play a role in explaining why we did not find an AVP effect on responses to cry sounds.

The current study has several limitations that should be addressed in future research. One important limitation is that conclusive information about the optimal dose of AVP and the best time interval between administration and behavioral effects is lacking. Based on the literature at hand it cannot be determined with certainty that our chosen dose and time interval were ideal. Also, due to the varying duration of other assessments, the exact time interval between hormone administration and the handgrip task varied somewhat among participants, which may have influenced the results. The exact mechanism of the effect of exogenous AVP on behavior, and the consequences for timing protocols, remain elusive and should be studied more extensively. Additionally, individual variation in the basal levels of, and sensitivity to, AVP might influence its behavioral effects, similar to what has been found for other hormones such as OT (see Bos, 2017 for a review), and future research may take these individual differences into account. Another important limitation is the sample size; we were only able to detect medium to large effects. Studies with larger sample sizes are warranted to confirm and extend these findings, preferably including participants in various phases of fatherhood (e.g., without children, early and late prenatal, early and late postnatal). Ultimately, more studies including physiological measures, such as was done here, are needed for the accumulation of knowledge about the (neuro)biological mechanisms underlying paternal care.

## Data Availability

The datasets generated for this study are available on request to the corresponding author.

## Ethics Statement

This study was carried out in accordance with the recommendations of ‘the Ethics Committees of the Institute for Education and Child Studies at Leiden University and the Leiden University Medical Centre, as well as the Dutch Central Committee on Research Involving Human Subjects’ with written informed consent from all subjects. All subjects gave written informed consent in accordance with the Declaration of Helsinki. The protocol was approved by the ‘the Ethics Committees of the Institute for Education and Child Studies at Leiden University and the Leiden University Medical Centre, as well as the Dutch Central Committee on Research Involving Human Subjects.’

## Author Contributions

MB-K designed and directed the project. KA-vD wrote the manuscript with support from AvV and MB-K. JW devised the auditory stimuli and JW and AvV programmed the task. KA-vD devised the visual stimuli. KA-vD, AvV, WM, AL, and JW performed the experiments. KA-vD, AvV, and AL analyzed the data. All authors provided critical feedback and helped shape the research and manuscript.

## Conflict of Interest Statement

The authors declare that the research was conducted in the absence of any commercial or financial relationships that could be construed as a potential conflict of interest.

## Footnotes

1 www.fantamorph.com
