# Supplementary material for: Vasopressin Differentially Affects Handgrip Force of Expectant Fathers in Reaction to Own and Unknown Infant Faces
Source: Front Behav Neurosci. 2019 May 21;13:105. doi: 10.3389/fnbeh.2019.00105 (PMC6536625; doi:10.3389/fnbeh.2019.00105)
Supplement: Supplementary file 1 [file Table_1.DOCX]

Supplementary Material

# Early Caregiving Experiences – Methods

Participants completed the Conflict Tactics Scale – Parent Child (CTS, Straus et al., 1998), which assesses experienced maltreatment during the participants’ childhood. We used items from the subscales Psychological aggression, Minor physical assault, Severe physical assault, and Neglect, resulting in a total of 18 items. Items were answered on a 7-point scale (0 =‘never’,1 = ‘once’, 2 = ‘twice’, 3 = ‘3–5 times’, 4 = ‘6–10 times’, 5 = ‘11–20times’, 6 = ‘more than 20 times’). Averaging scores on the minor and severe physical assault scales resulted in a Physical assault score, which combined with the scores on Psychological aggression formed an Abuse score. An overall CTS score was computed by averaging the Abuse and Neglect scales (M = 0.53, SD = 0.34), which was used for further analysis. One outlier (Z = 4.09) was winsorized to match the second highest score. The CTS total score was positively correlated with the Abuse scale, *r*= 0.92, *p* < .001, and with the Neglect scale, *r* = 0.53,*p* = .006. Additionally, participants completed seven items from the Withdrawal of Relations subscale of the Children’s Report of Parental Behavior Inventory (CRPBI, Beyers and Goossens, 2003; Schludermann and Schludermann, 1983) of which two items were slightly adapted for a smoother translation. To obtain a more comprehensive measurement of parental love-withdrawal, the questionnaire was complemented with four items from the Parental Discipline Questionnaire (PDQ, Patrick and Gibbs, 2007, see Huffmeijer et al., 2011 for the resulting scale). Participants rated how well each of the statements described their mother's or father's behavior on a 5-point scale ranging from 1 (not at all) to 5 (very well). A parental love-withdrawal score was computed by averaging the 11 maternal and 11 paternal scores (M = 1.72, SD = 0.47). The maternal and paternal scores correlated with the total score *r* = 0.74, *p* < .001, and *r* = 0.67, *p* < .001, respectively. See Thijssen et al., 2018 for use of the same questionnaires.

# Early Caregiving Experiences – Results

Correlations between mean handgrip force ratio’s and both CTS and parental love-withdrawal were found to be low (*r* ranging from .01 to .28), see Supplementary Table 1.

## Supplementary Table

## Supplementary Table 1. Correlation matrix for mean handgrip force ratio’s and early caregiving experiences.

|  | CRBI Love Withdrawal | CTS Abuse and Neglect |
| --- | --- | --- |
| Placebo – baseline | .072 | .088 |
| AVP – baseline | .176 | .040 |
| Placebo – own infant, control sound | .004 | -.107 |
| AVP – own infant, control sound | .119 | .017 |
| Placebo – own infant, cry sound | -.043 | -.127 |
| AVP – own infant, cry sound | .075 | -.009 |
| Placebo – unknown infant, control sound | .184 | .058 |
| AVP – unknown infant, control sound | .278 | -.127 |
| Placebo – unknown infant, cry sound | -.152 | -.050 |
| AVP – unknown infant, cry sound | .179 | .029 |
